# Simple Tube Centrifugation Method for Platelet-Rich Plasma (PRP) Preparation in Catalonian Donkeys as a Treatment of Endometritis-Endometrosis

**DOI:** 10.3390/ani11102918

**Published:** 2021-10-09

**Authors:** Priscila Fantini, Román Jiménez, Karina Vilés, Antoni Iborra, Maristela Silveira Palhares, Jaime Catalán, Marta Prades, Jordi Miró

**Affiliations:** 1 Department de Medicina i Cirurgia Animal, Universitat Autònoma de Barcelona, 08193 Cerdanyola del Vallès, Spain; fantinivet@yahoo.com.br (P.F.); romanfja@gmail.com (R.J.); vileskarina@gmail.com (K.V.); dr.jcatalan@gmail.com (J.C.); marta.prades@uab.cat (M.P.); 2 Departmento de Clínica e Cirurgia Veterinárias, Universidade Federal de Minas Gerais, Belo Horizonte 31270-901, Brazil; maristelapalhares@gmail.com; 3 SCAC Servei de Cultius Cellulars i Anticossos, Universitat Autònoma de Barcelona, 08193 Cerdanyola del Vallès, Spain; antoni.iborra@uab.cat

**Keywords:** donkey, platelet-rich plasma (PRP), ACD, TGF-β1

## Abstract

**Simple Summary:**

Platelet-rich plasma (PRP) is used to improve the regenerative capacity of damaged tissues in different species. In equine medicine, PRP is commonly used to treat joint diseases, tendinitis, ligamentous lesions, and persistent endometritis. Jenny endometrium shows a high sensitivity to endometritis. There are important differences between donkey and horse blood characteristics. Several protocols to obtain horse PRP have been reported, but no protocols have yet been reported for obtaining donkey PRP. Our study shows that single-spin tube centrifugation at 133× *g* for 10 min is appropriate to obtain donkey PRP with therapeutic potential in jenny endometritis-endometriosis.

**Abstract:**

The aim of this study was to standardize a simple, manual platelet-rich plasma (PRP) protocol in Catalonian donkeys using single-spin tube centrifugation as a treatment for jenny endometritis. The objective was to obtain a blood product with a moderate concentration of platelets (2 or 3 times baseline physiologic values) and a low WBC (White Blood Cells) concentration. Blood was drawn from six Catalonian donkeys using acid citrate dextrose (ACD) as an anticoagulant, and then processed by single centrifugation at 133× *g* for two different centrifugation times (10 and 15 min). The PRP samples were evaluated by flow cytometry, and TGF-β1 (Transforming Growth Factor-Beta1) concentrations were determined by enzyme-linked immunosorbent assay (ELISA). The 10 min centrifugation protocol resulted in a slightly greater release of TGF-β1 (6044.79 ng/mL), a 2.06-fold increase in platelet concentration, and a 15-fold reduction in leukocyte concentration when compared to the initial values. The 15 min centrifugation time resulted in a 2.44-fold increase in baseline platelet concentration, a reduction in WBC count by a factor of 20, and slightly lower TGF levels (5206 ng/mL). We conclude that both protocols are adequate for the obtention of PRP, and both may have an acceptable therapeutic potential for use in this species, although this needs to be further validated.

## 1. Introduction

Jenny endometrium shows high susceptibility to endometritis. Semen deposition induces an exacerbated inflammatory response, which is more pronounced than in mares [1,2]. Jenny endometritis is characterized by the presence of large amounts of polymorphonuclear neutrophils (PMNs) and eosinophils migrating to the uterine lumen, as well as increased cytokine expression [2,3].

After infection, neutrophils destroy pathogens by phagocytosis and by degranulation, producing DNA complexes that combine with nuclear and cytoplasmic proteins to form web-like extracellular structures called neutrophil extracellular traps (NETs) [4]. However, after intrauterine semen deposition, the PMN endometrial infiltration is induced by spermatozoa rather than bacteria [5]. A reduced number of spermatozoa are phagocytosed by PMNs, and most remain attached to NETs in a surrounding PMN halo [6]. NETs production is controlled by seminal plasma and represents the main sperm filter [7].

The failure of uterine drainage mechanisms in a mare can induce persistent metritis. NETs persistence has also been associated with the development of progressive fibrosis. Prostaglandins, inflammatory cytokines, and proteases found in NETs (e.g., elastase (ELA), cathepsin G (CAT), and myeloperoxidase (MPO)) act as pro-fibrotic factors in equine endometrial fibrosis by inducing collagen type I (COL1) accumulation [4,8,9,10].

Despite endometrial susceptibility, jenny endometritis seems to have its specific particularities, especially in the progression towards endometriosis. Inflammatory cells such as PMNs and eosinophils differ in timing and abundance, and there are also significant differences in the collagen distribution between jenny and mare [11].

Platelet-rich plasma is a platelet concentrate product that has been proposed as an essential agent to improve the regenerative capacity of damaged tissues [12], especially in the sub-acute phase of inflammation. Manual PRP preparation is fairly simple and inexpensive and consists of drawing whole blood in tubes with anticoagulants, then centrifuging the samples to obtain a greater concentration of platelets [13,14,15]. Because there are many different protocols used both in the human and veterinary literature and a lack of standardization in the realm of publications, the efficacy and treatment results are difficult to compare. Recently, Segabinazzi et al. [16] compared three different protocols to prepare equine PRP, obtaining no significant differences between one and two centrifugations, but did obtain a reduced platelet recovery using simple sedimentation. In the field of equine veterinary medicine, PRP is commonly used to treat degenerative joint diseases [17,18], tendinitis [19], ligamentous lesions [20,21], and persistent mating-induced endometritis (PMIE) [16,22,23].

Some of the most interesting molecules in PRP are the growth factors located within the platelet alpha granules (α). These factors play an important role in the modulation of inflammation and tissue repair since they have chemotactic properties and stimulate mitosis, proliferation, cellular differentiation [24], neovascularization, and extracellular matrix deposition [13,14,15,25]. There are several important growth factors in PRP, including platelet-derived growth factor (PDGF), transforming growth factors beta 1 and beta 2 (TGF-β1 and TGF-β2), epidermal growth factor (EGF), vascular endothelial growth factor (VEGF), insulin-like growth factor (IGF-1), and hepatocyte growth factor (HGF) [26,27]. TGF-β is considered a potent inflammation modulator [14,18,28,29] and analgesic agent since it inhibits the expression of nuclear factors and blocks the action of pro-inflammatory metabolites [14,30]. TGF-β1 is the most potent cytokine [31], and its concentration is used to monitor the increase in other growth factor concentrations [32].

One of the limitations of PRP is the lack of standardization of preparation. Consequently, the color, volume, and concentration of platelets, leukocytes, growth factors, and other cell types may vary from one preparation to another, which can lead to uneven and sometimes unsatisfactory clinical results [28,33,34,35]. It is also important to consider that blood characteristics vary according to species and breed [36]. Thus, in order to reduce the variability of the results and increase the rate of success, the PRP preparation protocol must be species-specific. There is no scientific literature available regarding PRP protocols in donkeys.

The aim of this study was to standardize a simple, manual, and inexpensive method of PRP preparation in donkeys while obtaining enough volume for the treatment of jenny endometritis-endometriosis.

## 2. Materials and Methods

**Figure 1:**
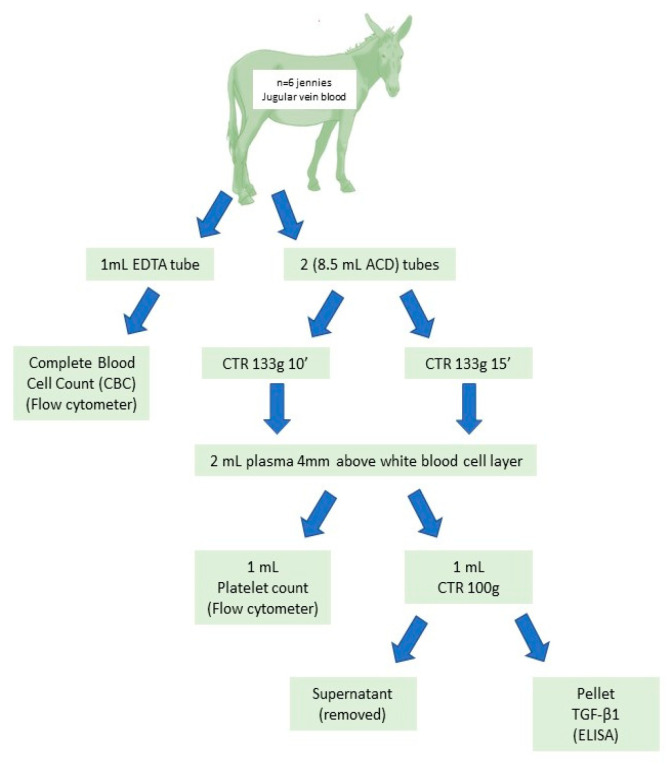
Experimental design.

This study was approved by the Ethics and Experimentation Committee of the Universitat Autónoma de Barcelona (CEUA/UFMG protocol number 294/2013). The experimental design is explained graphically in Figure 1.

### 2.1. Animals

Whole blood was collected from six Catalonian jennies, with ages varying from 6 to 14 years. The donkeys showed a good body condition and were clinically healthy based on physical examination and a complete blood cell analysis. Jennies were grouped in a big paddock at the Equine Reproduction Service, Autonomous University of Barcelona (Bellaterra, Cerdanyola del Vallés, Spain). Animals were fed grain forage, straw, and hay, and water was provided ad libitum.

### 2.2. PRP Preparation

The blood was collected through an aseptic puncture of the jugular vein, using a Vacutainer (Venosafe, Leuven, Belgium) system and a 23 G needle. A sample of the blood was taken and transferred to a 1 mL EDTA (Ethylenediaminetetraacetic acid) tube for a complete blood cell count (CBC), while the remaining blood was transferred to two 8.5 mL tubes (tubes 1 and 2), containing 1.5 mL of ACD (Anticoagulant Citrate Dextrose solution) anticoagulant (Trisodium citrate at 22 g/L, citric acid at 8.0 g/L, and dextrose at 24.5 g/L) (Becton Dickinson, Franklin Lakes, NJ, USA). In sequence, the blood was homogenized manually through slow inversions of the tube.

Several preparation protocols of PRP used in horses were tested in donkeys (non-published data), and the most interesting results were obtained via the simple tube centrifugation method. The following protocol was selected based on reproducibility and ease of preparation. The tubes were centrifuged (CTR) at 133× *g* for two different centrifugation times, tube 1 being centrifuged for 10 min and tube 2 centrifuged for 15 min. In each tube, 2.0 mL of the plasma fraction was collected in a laminar flow chamber using an 18 G spinal needle (Becton Dickinson, Madrid, Spain). The tip of the needle was introduced until approximately 4 mm above the white blood cell layer (buffy coat) in order to recover the maximal number of platelets and the minimum possible amount of leukocytes.

Once the PRP was prepared, a 1.0 mL sample was submitted for a platelet count and evaluation by flow cytometry at the Veterinary Hematology Service of the Universitat Autònoma de Barcelona. The remaining PRP was then centrifuged at 1000× *g* to promote platelet sedimentation. After centrifugation, the supernatant was removed and immediately frozen and stored at −20 °C. These samples were submitted for ELISA testing to determine the TGF-β1 concentrations (SCAC, Servei de Cultius Celulars i Anticossos, Universitat Autònoma de Barcelona).

### 2.3. Platelet Count and Evaluation

Complete blood cell counts (CBCs) were performed by a hematological cytometer (ADVIA 120^®^ Siemens Medical Solutions; Fernwald, Germany), which is a flow cytometer with two laser beams, making it possible to contrast the same value and generate accurate and reliable results. The leukocyte, erythrocyte, and platelet panels were then evaluated. The platelet parameters studied included platelet count (PLT), plateletcrit (PCT), mean platelet volume (MPV), mean platelet component concentration (MPC), platelet volume of distribution width (PDW), mean platelet dry mass (MPM), platelet dry mass distribution width (PMDW), platelet component distribution width (PCDW), concentration of large platelets (LAPLT), and platelet clumps (CLUMPS).

### 2.4. Growth Factor Concentration

TGF-β1 (Transforming Growth Factor-Beta1) was measured by ELISA, using commercially available antibodies (DuoSet ref. DY240, Bayer Lab, New York, NY, USA). The TGF-β1 concentration was determined using a mouse antigen-catching antibody specific for human TGF-β1 and a biotinylated chicken detection antibody, also specific for human TGF-β1. Human TGF-β1 recombinant patterns were also used to elaborate the standard curve. The use of this kit in horses has already been tested and approved in previous studies of this research group [14], but its use in donkeys had not been tested. Samples were analyzed in duplicates, showing no significant differences. Observed differences were lower than previously reported in horses [14].

The samples were activated to quantify the growth factors by ELISA. This activation triggered the release of latency-associated peptides and protein disulfide isomerase (PDI) and, consequently, the formation of the inactive TGF-β1 complex, secreted by the cells. The activation was performed in vitro by the addition of 100 µL of activation buffer (acetic acid 2.5 N (Ref. 010.520, Sigma-Aldrich, Barcelona, Spain) and urea 10 M (Ref. U-5128, Sigma-Aldrich, Barcelona, Spain) to the same volume of plasma, followed by homogenization and incubation at room temperature for 10 min. The pH was neutralized with 100 µL of neutralizing buffer (NaOH 2.7 M (Ref. 141687.1211, Panreac, Barcelona, Spain)/HEPES 1 M (Ref. H3375, Sigma-Aldrich, Barcelona, Spain). We obtained different dilutions of the samples, activated with the dilution buffer (bovine serum albumin 1.4%, Ref. 10735078001, Roche, Barcelona, Spain), Tween 20 0.05% (Ref. 170-6531, Bio-Rad, Barcelona, Spain), in PBS (Phosphated Buffered Saline).

### 2.5. Statistical Analysis

The data were analyzed with commercial software (SPSS® Ver. 25.0 for Windows; IBM Corp., Armonk, NY, USA) and expressed as means ± standard errors. Single-factor analysis of variance (ANOVA) was used for statistical comparisons, while the post hoc comparisons were analyzed through a Tukey’s test to determine the differences between means with normal distribution, as well as variables that presented a normal distribution after log (x + 1) transformation. The variables that did not present a normal distribution after the logarithmic transformation were analyzed through the Student’s t-test, and the post hoc comparisons were made through the Wilcoxon test. Differences were considered statistically significant when the *p*-values were <0.05.

## 3. Results

### 3.1. Blood Cell Count and Growth Factors Determination

Whole blood and PRP contained different values (*p* < 0.05) of PLT, MPV, MPC, PCDW, CLUMPS, WBC White Blood Cells), RBC (Red Blood Cells), and PMN. However, there were no differences in these values (*p* > 0.05) between the PRPs prepared with different protocols. The TGF-β1 concentration was greater in the protocol with a shorter centrifugation time (Table 1).

### 3.2. Preliminary “In Vivo” Experience

Two Catalonian jennies, 15 and 17 years old, with endometrial biopsies categorized as IIB in Kenney and Doig classification and presenting infertility for the last two years, were treated once. An intrauterine infusion was performed during estrus with 20 mL of PRP (133× *g* for 10 min) with success. The following estrus, both were inseminated and became pregnant.

## 4. Discussion

No studies were found in the literature regarding PRP preparation protocols in donkeys. This is the first study evaluating PRP preparation in donkeys, including the measurement of TGF-β1 concentrations with different protocols. The hematological parameters of whole blood were consistent with previous parameters described for Catalonian donkeys [37]. The ADVIA 120^®^ enabled a more accurate platelet count when compared to conventional automatized hematological analyzers, providing parameters that can be related to the activation status of the platelets. Both products showed platelet activation evidenced by an MPV (size) increase, and decreases in MPC (density) and PCDW (platelet component distribution width). The 15 min protocol showed a slight decrease in TGF-β1 concentrations, and this is consistent with studies in other species which show that there may be variability in TGF beta yield even within the same individual at different sampling times [38].

There were no differences (*p* < 0.05) in the platelet counts between centrifugation for 10 and 15 min. The tubes centrifuged at 133× *g* for 10 min presented a 2.06-fold increase in platelet concentration and a reduction in leukocyte concentration by a factor of 15 compared to initial values, whereas the tubes centrifuged at the same gravitational force for 15 min presented a 2.44-fold increase in platelet concentration and a 20 times reduction in leukocyte concentration. In accordance with the classification proposed by Dohan Ehrenfest et al. [39], the platelet concentrates that have 1.3 to 3 times the initial platelet value and a leukocyte concentration similar to or lower than the concentration in the whole blood are known as pure platelet-rich plasma (P-PRP). Thus, the PRP obtained can be considered a P-PRP. The mean values of platelets yielded from donkeys’ blood were higher than the values reported in equine blood samples submitted to double tube centrifugation [14,40]. Basal donkey platelet counts are higher than those in horses [37].

In the 10 min protocol, platelet activation was less apparent than in samples centrifuged for 15 min, as expressed by a lower number of CLUMPS, lower MPV, lower PCDW, and a higher MPC. However, the differences were not statistically significant (*p* > 0.05). The large individual variation, characterized by the standard error of the mean and coefficient of variation, may indicate the necessity of including a greater number of animals in future research.

It is known that the portion of the plasma with the highest platelet concentration is the 2 mm fraction above the buffy coat [13,27]. However, it was decided to collect the plasma fraction 4 mm above the buffy coat in an attempt to recover the lowest possible number of leukocytes, in order to avoid an increase in catabolic signaling molecules [41].

The higher concentration of platelet clumps in the whole blood may be due to the EDTA, since this anticoagulant is capable of producing structural and functional damage in platelets [42,43].

The higher TGF-β1 concentration in the 10 min protocol (*p* < 0.05), even with a lower platelet concentration, may be due to the higher concentration of viable non-activated platelets in this protocol compared to the 15 min protocol. It is necessary to conduct more studies regarding platelet activation since, according to some authors, a higher number of viable non-activated platelets is desired [44,45,46] in order to have a more prolonged effect on growth factor release in time, although this has yet to be proven.

Several in vivo and in vitro experiments have shown beneficial effects of platelet-related products in the modulation of the inflammatory process and tissue healing. To optimize PRP composition and facilitate its preparation in other species, several methods have been described. Although the literature in PRP is extensive, the lack of evidence and consensus about what method or PRP composition to use in different diseases or tissue trauma remains. In general, manual methods intended for PRP production present lower costs, but may be time-consuming and require a strict aseptic technique. Since the method described here is based on one single spin with an acceptable end product that was validated clinically in a few animals, we have provided the first step to finding the ideal product in donkeys with a method that is simple and cost-effective.

## 5. Conclusions

The methodology employed for the PRP preparation in donkeys with single tube centrifugation at 133× *g* for 10 min was appropriate to obtain supraphysiological concentrations of platelets with a significant and concurrent reduction in leukocyte content. With this protocol, the PRP also presented an acceptable concentration of TGF-β1 as a growth factor sentinel, possibly providing enough volume and therapeutic potential for use in damaged jenny endometrium. It should be noted that there are important differences between jenny and mare endometritis and endometriosis [11], and that platelets and cytokine concentrations showed different effects (positive or adverse) in other tissues [41,47,48,49]. Further studies are necessary to optimize the jenny endometritis-endometriosis treatment by PRP.

## Figures and Tables

**Table 1:** Plasma components of whole-blood samples (EDTA) and PRP (ACD) obtained by centrifugation at 133× *g* for 10 and 15 min.

Variable	Whole Blood EDTA	PRP 133× *g*/10 min	PRP 133× *g*/15 min
PLT (×10^3^/µL)	196.66 ± 51.42 ^b^	406.5 ± 27.29 ^a^	479.50 ± 75.05 ^a^
MPV (fL)	8.38 ± 0.44 ^b^	11.25 ± 0.27 ^ab^	15.05 ± 2.6 ^a^
MPC (g/dL)	22.35 ± 1.32 ^a^	17.53 ± 0.21 ^b^	13.55 ± 2.15 ^b^
PCDW (g/dL)	7.58 ± 0.22 ^a^	7.16 ± 0.1 ^b^	5.20 ± 1.14 ^b^
LAPLT (×10^3^ cells/µL)	8.161 ± 3.29 ^a^	35.16 ± 5.35 ^a^	154.50 ± 85.64 ^a^
RBCFRAG (×10^6^ cells/µL)	0.02 ± 0.006 ^a^	0.04 ± 0.003 ^ab^	0.10 ± 0.03 ^a^
CLUMPS	1196.83 ± 390.15 ^a^	83.66 ± 11.35 ^b^	89.00 ± 38.28 ^b^
WBC (×10^3^ cells/µL)	10.86 ± 0.80 ^a^	0.74 ± 0.16 ^b^	0.52 ± 0.32 ^b^
RBC (×10^6^ cells/µL)	6.56 ± 0.25 ^a^	0.05 ± 0.004 ^b^	0.16 ± 0.07 ^b^
PMN (×10^3^ cells/µL)	5.12 ± 0.40 ^a^	0.15 ± 0.005 ^b^	0.16 ± 0.12 ^b^
TGF-β1 (pg/mL)	-	6044.79 ± 417.91 ^a^	5206.88 ± 289.20 ^b^
PROT (pg/mL)	-	876.03 ± 112.65 ^a^	661.47 ± 66.27 ^b^
TGF:PROT	-	14.68 ± 1.64 ^a^	12.79 ± 1.25 ^b^

PLT, platelet count; MPV, mean platelet volume; MPC, mean platelet component concentration; PCDW, platelet component distribution width; LAPLT, large platelets; RBCFRAG, fragments of red blood cells; CLUMPS, platelet clumps; WBC, white blood cells; RBC, red blood cells; NEU, neutrophils; TGF-β1, transforming growth factor-beta 1; PROT, total protein. Different letters in the same line represent significantly different values (*p* < 0.05).

## Data Availability

Obtained data averages are exposed in the article.
